# A proposed simplified definition of metabolic syndrome in children and adolescents: a global perspective

**DOI:** 10.1186/s12916-024-03406-y

**Published:** 2024-05-07

**Authors:** Xin’nan Zong, Roya Kelishadi, Hae Soon Kim, Peter Schwandt, Tandi E. Matsha, Jose G. Mill, Carmelo Antonio Caserta, Carla Campos Muniz Medeiros, Anastasios Kollias, Peter H. Whincup, Lucia Pacifico, Abel López-Bermejo, Min Zhao, Miaobing Zheng, Bo Xi

**Affiliations:** 1 Department of Growth and Development, Capital Institute of Pediatrics, Beijing, China; 2 Department of Epidemiology, School of Public Health, Qilu Hospital, Cheeloo College of Medicine, Shandong University, Jinan, China; 3 Child Growth and Development Research Center, Research Institute for Primordial Prevention of Non Communicable Disease, Isfahan University of Medical Sciences, Isfahan, Iran; 4 Department of Pediatrics, Ewha Womans University College of Medicine, Seoul, Korea; 5 Atherosclerosis Prevention Institute, Munich-Nuremberg, Munich, Germany; 6 Department of Biomedical Sciences, Faculty of Health & Wellness Sciences, Cape Peninsula University of Technology, Cape Town, South Africa; 7 Department of Physiological Sciences, Center of Health Sciences, Federal University of Espírito Santo, Vitória, Brazil; 8 Associazione Calabrese Di Epatologia - Medicina Solidale - A.C.E. ETS, Reggio Calabria, Italy; 9 Department of Public Health, State University of Paraiba, Campina Grande, Brazil; 10 Hypertension Center STRIDE-7, School of Medicine, Third Department of Medicine, National and Kapodistrian University of Athens, Sotiria Hospital, Athens, Greece; 11 Population Health Research Institute, St George’s, University of London, London, UK; 12 Department of Maternal and Child Health, Sapienza University of Rome, Rome, Italy; 13 Pediatric Endocrinology Research Group, Girona Biomedical Research Institute (IDIBGI), Salt, Spain; 14 Department of Pediatrics, Hospital Dr. Josep Trueta, Girona, Spain; 15 Department of Medical Sciences, University of Girona, Girona, Spain; 16 Department of Nutrition and Food Hygiene, School of Public Health, Shandong University, Jinan, China; 17 Institute for Physical Activity and Nutrition, School of Exercise and Nutrition Sciences, Deakin University, Geelong, Australia

**Keywords:** Metabolic syndrome, Central obesity, Hypertension, Cardiovascular risk factors, Child, Waist-to-height ratio, Adolescent

## Abstract

Metabolic syndrome (MetS) is becoming prevalent in the pediatric population. The existing pediatric MetS definitions (e.g., the International Diabetes Federation (IDF) definition and the modified National Cholesterol Education Program (NCEP) definition) involve complex cut-offs, precluding fast risk assessment in clinical practice.

We proposed a simplified definition for assessing MetS risk in youths aged 6–17 years, and compared its performance with two existing widely used pediatric definitions (the IDF definition, and the NCEP definition) in 10 pediatric populations from 9 countries globally (*n* = 19,426) using the receiver operating characteristic (ROC) curve analyses. In general, the total MetS prevalence of 6.2% based on the simplified definition was roughly halfway between that of 4.2% and 7.7% estimated from the IDF and NCEP definitions, respectively. The ROC curve analyses showed a good agreement between the simplified definition and two existing definitions: the total area under the curve (95% confidence interval) of the proposed simplified definition for identifying MetS risk achieved 0.91 (0.89–0.92) and 0.79 (0.78–0.81) when using the IDF or NCEP definition as the gold standard, respectively.

The proposed simplified definition may be useful for pediatricians to quickly identify MetS risk and cardiometabolic risk factors (CMRFs) clustering in clinical practice, and allow direct comparison of pediatric MetS prevalence across different populations, facilitating consistent pediatric MetS risk monitoring and the development of evidence-based pediatric MetS prevention strategies globally.

**Supplementary Information:**

The online version contains supplementary material available at 10.1186/s12916-024-03406-y.

## Key messages

• There is no consensus on definition for assessing pediatric metabolic syndrome (MetS). The existing pediatric MetS also involve age-, sex- or height- specific percentile cut-offs for some components (e.g., central obesity and elevated blood pressure), hindering quick assessment of MetS risk and cardiometabolic risk factors (CMRFs) clustering in clinical practice.

• The proposed simplified definition of MetS for children and adolescents aged 6–17 years was developed based on two widely used IDF and NCEP definitions with inclusion of simple static cut-offs emerged from the recent literature, and with involvement of two risk assessment levels (monitoring and action levels) to assess severity of MetS risk.

• The proposed simplified definition showed a good performance in identifying youths with MetS risk when compared with the IDF and NCEP definitions in a diverse sample of 10 pediatric populations globally. Moreover, the simplified definition involves static cut-offs instead of complex cut-offs for each component, supporting quick assessment and easy application in clinical practice.

• The proposed simplified definition may be useful for pediatricians to quickly identify MetS risk and CMRFs clustering in clinical practice, and allow direct comparison of pediatric MetS prevalence across different populations, facilitating consistent pediatric MetS risk monitoring and the development of evidence-based pediatric MetS prevention strategies globally.

**Supplementary Information:**

The online version contains supplementary material available at 10.1186/s12916-024-03406-y.

## Background

Metabolic syndrome (MetS) is defined as a cluster of cardiometabolic risk factors (CMRFs), including central obesity, high blood pressure (BP), dyslipidaemia, and high fasting glucose [1]. Compelling evidence suggests that early onset of MetS has become more common and childhood MetS can track into adulthood, thereby increasing the risk of future cardiovascular disease. In 2020, about 3% of children aged 6–12 years, and 5% of adolescents aged 13–18 years had MetS globally [2]. Notably, several definitions have been used to define pediatric MetS but no consensus on the MetS definition has been established. The existing widely used pediatric MetS definitions such as the International Diabetes Federation (IDF) definition and the modified National Cholesterol Education Program (NCEP) definition involve different definitions and heterogenous cut-offs for MetS components [3, 4], impeding pediatricians to quickly assess MetS risk and CMRFs clustering in clinical practice. For example, age- and sex- specific waist circumference (WC) percentile cut-offs are used for defining central obesity in both IDF and NCEP definitions. Likewise, age-, sex- and height- specific systolic/diastolic blood pressure (SBP/DBP) percentile cut-offs are used to define elevated BP in NCEP definition. Thus, development of an easy-to-apply and standardized definition to facilitate the clinical diagnosis of pediatric MetS is imperative.

Emerging evidence has showed the potential of using simplified static cut-offs for defining central obesity and elevated BP in children and adolescents. For instance, several systematic reviews showed that waist-to-height ratio (WHtR) is a useful alternative for WC to predict pediatric MetS and identify youths with CMRFs clustering [5, 6]. Indeed, WHtR has already been proposed for assessing central obesity as a component of pediatric MetS definition [7–9]. Furthermore, a recent pooled analysis of 34,224 children and adolescent aged 6–18 years from multiple countries reported that simplified WHtR cut-offs (i.e., 0.50 for European and US youths, and 0.46 for Asian, South American and African youths) were robust for identifying central obesity in children and adolescents [10]. In addition, the simplified static cut-offs of SBP/DBP (i.e., 120/80 mm Hg for children aged 6–12 years and 130/80 mm Hg for adolescents aged 13–17 years) have also been widely validated and recommended to define elevated BP in the pediatric population [11–14]. By incorporating these simple static cut-offs, we proposed a new simplified definition of pediatric MetS and validated its performance in 10 pediatric populations from 9 countries worldwide.

## Main text

### The proposed simplified definition of pediatric MetS

The proposed simplified definition of MetS for children and adolescents aged 6–17 years was developed based on two widely used definitions of pediatric MetS (the IDF definition [11] and the NCEP definition) [15], with static cut-offs for defining all five components. The simplified definition defines MetS as the presence of three or more of the five components (i.e., central obesity, high BP, high triglycerides (TG), low high-density lipoprotein cholesterol (HDL-C), and high fasting blood glucose (FBG)) without specifying central obesity as an essential component. Of note, for either the IDF or NCEP definition, central obesity was defined as WC ≥ international age- and sex- specific 90th percentile values [16]. For the NCEP definition, high BP was defined as SBP/DBP ≥ international age-, sex- and height- specific 90th percentile values [17]. In the proposed simplified definition, we replaced age-, sex-, or height-specific percentile cut-offs of WC or SBP/BDP in the IDF or NCEP definition by simplified static cut-offs emerged from recent literature, and updated cut-offs for defining dyslipidemia and high fasting glucose. That is, central obesity was defined as WHtR ≥ 0.50 for youths from Europe and the USA and ≥ 0.46 for those from Asia, Africa, and South America [10]; high BP was defined as SBP/DBP ≥ 130/80 mm Hg for adolescents aged 13–17 years [13] and ≥ 120/80 mm Hg for children aged 6–12 years [12, 14]; high TG was defined as TG ≥ 130 mg/dl at age 10–17 years or ≥ 100 mg/dl at age 6–9 years [18], or low HDL-C as HDL-C < 40 mg/dl [11, 15]; and high FBG was defined as FBG ≥ 100 mg/dl [11, 19]. A comparison of cut-offs of five individual components for the simplified definition and the IDF and NCEP definitions is shown in Table 1.

**Table 1:** Cut-offs of metabolic syndrome components based on three definitions in children and adolescents

MetS definition	Level	Central obesity	High BP	Dyslipidaemia	High fasting glucose
IDF [11]^a^		WC ≥ 90th percentile values [16]	SBP/DBP ≥ 130/85 mm Hg	TG ≥ 150 mg/dl, or HDL-C < 40 mg/dl (< 50 mg/dl for girls aged ≥ 16 years)	FBG ≥ 100 mg/dl
NCEP [15]^b^		WC ≥ 90th percentile values [16]	SBP/DBP ≥ 90th percentile values [17]	TG ≥ 110 mg/dl, or HDL-C ≤ 40 mg/dl	FBG ≥ 110 mg/dl
Simplified definition^c^	Monitoring level				
		WHtR ≥ 0.50 for youths from Europe and the USA or ≥ 0.46 for youths from Asia, Africa and South America [10]	SBP/DBP ≥ 130/80 mm Hg at age 13–17 years [13], or ≥ 120/80 mm Hg at age 6–12 years [12, 14]	TG ≥ 130 mg/dl at age 10–17 years or ≥ 100 mg/dl at age 6–9 years [18], or HDL-C < 40 mg/dl [11, 15]	FBG ≥ 100 mg/dl [11, 19]
	Action level				
		WHtR ≥ 0.55 in youths from Europe and USA or ≥ 0.50 in those from in Asia, Africa and South America [20, 21]	SBP/DBP ≥ 135/85 mm Hg for adolescents aged 13–17 years or ≥ 125/85 mm Hg for children aged 6–12 years	TG ≥ 150 mg/dl at age 10–17 years [11] or ≥ 110 mg/dl at age 6–9 years [15], or HDL-C < 35 mg/dl [22, 23]	FBG ≥ 110 mg/dl [15]

*Abbreviations DBP* diastolic blood pressure, *FBG* fasting blood glucose, *HDL-C* high-density lipoprotein cholesterol, *IDF* International Diabetes Federation, *NCEP* National Cholesterol Education Program, *SBP* systolic blood pressure, *TG* triglycerides, *WC* waist circumference, *WHtR* waist-to-height ratio

^a^The IDF definition is recommended for youths aged 10–17 years

^b^The NCEP definition is recommended for youths aged 12–19 years

^c^The simplified definition is recommended for youths aged 6–17 years

### Validation of the performance of the proposed simplified MetS definition in 10 pediatric populations

It should be noted that the IDF definition was recommended for youths aged 10–17 years and the NCEP was for those aged 12–19 years, but the proposed simplified definition is recommended for youths aged 6–17 years. In the following validation study, we just focused on the validation in adolescents aged 12–17 years for direct comparison with the IDF and NCEP definitions.

We firstly searched relevant studies on pediatric MetS or metabolic risk factors in PubMed database and we then invited the corresponding author of each study to participate in this work. Finally, individual data on metabolic risk factors globally, including 15 pediatric populations aged 6–18 years from Africa, Asia, Europe, North America and South Africa [10] were available for making the contribution. Overall, 19,426 adolescents (boys: 50.8%) aged 12–17 years, with complete data on sex, age, height, weight, WC, SBP, DBP, TG, HDL-C, and FBG, contributed to this present study. The characteristics of 10 pediatric populations from 9 countries (i.e., Brazil, China, Germany, Greece, Iran, Italy, Korea, South Africa, and the USA) have been described elsewhere [10]. In brief, the 10 study populations included six population-based cross-sectional surveys including eighteen public high schools in Northeastern Brazil (Brazil_a, 2012–2013), a community project (Estação Conhecimento) in Vitória, Brazil (Brazil_b, 2014–2016), a community-based Praeventions-Erziehungs-Programm (PEP) Family Heart Study in Germany (2000–2007), a survey in five schools in the Karlovassi province of Greece (2008–2010), a survey of eight primary schools in Calabria, Italy (2007–2008), and a school-based survey in South Africa (2007–2008), as well as four nationally representative surveys including the China Health and Nutrition Survey (CHNS, 2009), the Childhood and Adolescence Surveillance and Prevention of Adult Non-communicable Diseases in Iran (2011–2012), the Korean National Health and Nutrition Examination Surveys (2001–2013), and the US National Health and Nutrition Examination Surveys (NHANES, 2001–2018). WHtR was calculated as WC (cm)/height (cm). Each study received ethical approval from respective institutional review boards, and informed consent from the study participants and their parents/guardians.

We compared the prevalence of MetS across 10 pediatric populations by three definitions: the IDF definition, the NCEP definition and the simplified definition using the Chi-square test. The performance (accuracy) of the simplified definition in identifying MetS (yes vs no) using either IDF or NCEP definition as the gold standard was assessed by the receiver operating characteristic (ROC) curve analyses, with the calculation of area under the curve (AUC), sensitivity, specificity, positive predictive value (PPV), and negative predictive value (NPV). Generally, an AUC value < 0.7 is considered poor, 0.7–0.8 as acceptable and > 0.8 as good [24]. Basic data analyses were undertaken using SAS 9.4 (SAS Institute, Cary, NC). The ROC curve analyses were performed using reportROC 3.6 package running under R 4.2.2. Two-tailed *P* < 0.05 were considered statistical significance. As the IDF definition specified central obesity as an essential component, we also conducted additionally a sensitivity analysis to test if specifying central obesity as an essential component would alter the estimation of MetS prevalence by the simplified definition.

**Fig. 1:**
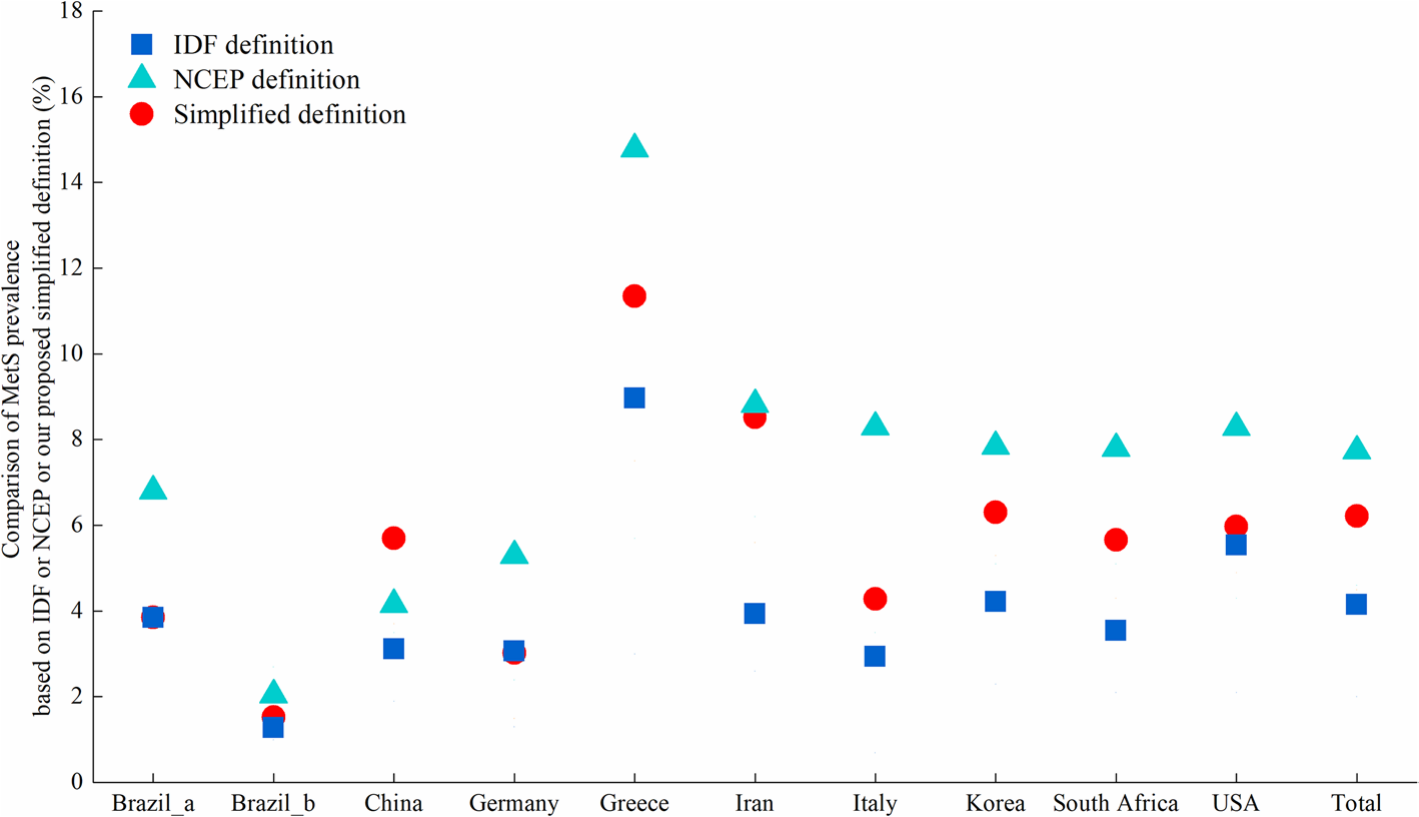
Comparison of metabolic syndrome prevalence based on the simplified definition, IDF definition and NCEP definition in 10 pediatric populations

In general, the total MetS prevalence (6.2%) across 10 populations estimated by the simplified definition was roughly halfway between the prevalence estimated by the IDF (4.2%) and NCEP (7.7%) definitions (Fig. 1 and Additional file 1: Table S1&S2).

The ROC curve analyses showed that the total AUC (95% CI) of the simplified definition for identifying MetS in the 10 study populations achieved 0.91 (0.89–0.92) and 0.79 (0.78–0.81) when using the IDF or NCEF definition as the gold standard, respectively (Table 2).

**Table 2:** Performance of the simplified definition for identifying metabolic syndrome risk using IDF definition or NCEP definition as gold standard

Country	AUC (95% CI)	Sensitivity	Specificity	PPV	NPV
*Performance of simplified definition using IDF definition as gold standard*					
Brazil_a	0.878 (0.772–0.983)	0.765	0.991	0.765	0.991
Brazil_b	0.999 (0.996–1.000)	1.000	0.997	0.833	1.000
China	0.987 (0.978–0.995)	1.000	0.973	0.545	1.000
Germany	0.782 (0.727–0.836)	0.576	0.987	0.583	0.987
Greece	0.906 (0.836–0.976)	0.853	0.959	0.674	0.985
Iran	0.910 (0.884–0.936)	0.873	0.947	0.403	0.995
Italy	0.899 (0.778–1.000)	0.818	0.981	0.562	0.994
Korea	0.944 (0.924–0.965)	0.914	0.974	0.612	0.996
South Africa	0.915 (0.852–0.978)	0.857	0.973	0.536	0.995
USA	0.900 (0.870–0.930)	0.816	0.985	0.756	0.989
Total	0.905 (0.891–0.919)	0.838	0.972	0.561	0.993
*Performance of simplified definition using NCEP definition as gold standard*					
Brazil_a	0.783 (0.695–0.872)	0.567	1.000	1.000	0.969
Brazil_b	0.747 (0.571–0.924)	0.500	0.995	0.667	0.990
China	0.894 (0.791–0.998)	0.812	0.976	0.591	0.992
Germany	0.674 (0.634–0.715)	0.361	0.988	0.631	0.965
Greece	0.737 (0.661–0.814)	0.518	0.957	0.674	0.920
Iran	0.822 (0.797–0.846)	0.672	0.972	0.696	0.968
Italy	0.740 (0.650–0.831)	0.484	0.997	0.938	0.955
Korea	0.821 (0.796–0.845)	0.655	0.987	0.815	0.971
South Africa	0.737 (0.677–0.797)	0.494	0.980	0.679	0.958
USA	0.786 (0.755–0.817)	0.585	0.988	0.810	0.963
Total	0.791 (0.778–0.805)	0.600	0.983	0.747	0.967

*Abbreviations AUC* area under the curve, *IDF* International Diabetes Federation, *CI* confidence interval, *NCEP* National Cholesterol Education Program, *NPV* negative predictive value, *PPV* positive predictive value, *USA* United States of America

Sensitivity analyses showed that the total MetS prevalence of 6.2% based on the simplified definition without central obesity as an essential component was slightly higher than that of 5.6% with central obesity as an essential component (Additional file 1: Table S3). This suggested that not specifying central obesity as an essential component in pediatric MetS definition may be better in terms of avoiding potential missed diagnosis of youths with MetS risk.

## Discussion

To our knowledge, we proposed the first simplified and easy-to-apply definition for assessing MetS in pediatric population. The simplified definition also demonstrated good performance in identifying youths with MetS risk when compared with two widely used pediatric MetS definitions (i.e., the IDF definition, the NCEP definition) in 10 diverse pediatric populations globally.

Due to discrepant cut-offs are used for defining MetS components, the prevalence estimations of MetS varied greatly between the IDF and NCEP definitions. The variability of MetS prevalence across the populations may be mainly influenced by the nutritional status of the populations, with the prevalence of overweight&obesity ranging from 10.1% in China to 39.8% in Greece across the 10 populations according to BMI categories using Cole’s cut-off points [25] (Table S1). Additionally, other factors such as demographic characteristics, geographic location, and socioeconomic status may also influence the variability of MetS prevalence across the populations. The IDF definition tends to estimate lower prevalence of MetS than that of the NCEP definition. However, the MetS prevalence estimated by the simplified definition was within the prevalence estimations defined by IDF and NCEP definitions, which seemed to support the validity of the simplified definition. Moreover, the simplified definition also has several advantages over the existing MetS definitions. The utilization of simple and static cut-offs in lieu of complex age- and sex-specific cut-offs for defining each MetS component could facilitate easy application in clinical practice. The ROC curve analyses showed that the simplified definition was highly consistent with both IDF and NCEP definitions for estimating MetS prevalence with AUC ranging from 0.79 to 0.91. Whether using the IDF or NCEP definition as the gold standard, the simplified definition showed high specificity and NPV, which means its high ability in identifying non-MetS children. However, the sensitivity and PPV seemed a little lower, which suggests that potential MetS children can be identified by the simplified definition but they may require further diagnosis. It is encouraging that replacing complex cut-offs (i.e., age- and sex- specific WC percentile values for defining central obesity, and additional height- specific BP percentile values for defining high BP) with simple static cut-offs did not appear to sacrifice the performance or accuracy in identifying pediatric MetS risk.

The existing widely used pediatric MetS definitions are intended for use among children aged 10 years or older. For instance, the IDF and NCEP definitions were designed for use in youths aged 10–17 years and 12–19 years, respectively. It is well-documented that metabolic abnormalities such as insulin resistance and dyslipidaemia are already prevalent in prepubertal children aged 10 years and under [26, 27]. A recent prospective cohort study showed that childhood CMRFs were positively associated with adulthood cardiovascular events [28]. Another longitudinal study showed that controlling obesity and related CMRFs during the prepubertal stage appeared to be critical in preventing pubertal MetS effectively [27]. Additionally, a recent systematic review suggested the importance of initiating the prevention of atherosclerosis in early life [29]. Considering the increasing prevalence of MetS and CMRFs clustering in prepubertal children and its far-reaching health implications [2, 30], early diagnosis of the MetS among prepubertal children is also warranted [31]. The proposed simplified definition addresses this gap by enabling MetS risk assessment for both prepubertal and pubertal children from ages 6 to 17 years. However, the performance of the simplified definition can not be validated for children aged 6–11 years in current study because of unavailability of gold standard in this specific age group. In addition, our proposed simplified definition used static cut-offs for defining central obesity and elevated BP, which is very convenient and easy-to-apply for rapid screening in clinical practice compared with two widely used existing definitions (e.g., IDF or NCEP definition).

Apart from developing simplified ‘monitoring level’ definition for MetS risk monitoring at conservative population level, we also proposed a ‘action level’ definition with more stringent cut-offs to guide pediatric clinical practice to identify severely affected youths who require an immediate intervention. The ‘action level’ definition also includes meeting at least 3 of the same 5 components, but the cut-offs for defining the 5 components are set more stringently. In our pooled population, the total MetS prevalence estimates at ‘monitoring level’ and ‘action level’ were 6.2% and 1.2% in adolescents aged 12–17 years, respectively. The development of both monitoring and action level definitions may be better to guide clinical practice for identifying severity of MetS risk [32]. The monitoring level definition identifies at-risk youths who requires close monitoring and observation whereas the action level identifies severely at-risk youths who require a timely intervention to ameliorate the risk profile. It is potentially useful for applications of simplified pediatric MetS definition in clinical practice for early detection of MetS, risk stratification, and targeted interventions.

A recent review commented that developing a consistent global definition of pediatric MetS currently faced several challenges, including the variations in child anthropometric and metabolic characteristics by race/ethnicity or geographic regions or pubertal stages, and a single definition can not differentiate the severity of MetS risk [3]. In the process of developing the simplified definition, we attempted to overcome these challenges by incorporating specific WHtR cut-offs for different racial/ethnic groups and geographic areas), and different cut-offs of BP or TG for younger children and older adolescents accounting for pubertal stages. Furthermore, we developed two level definitions (monitoring and action levels) to assess severity of MetS, as recommended by the American Heart Association [33].

### Limitations

First, although we conducted a large-scale validation for the simplified definition in a diverse mixed sample of adolescents aged 12–17 years from 9 countries, further validation in more geographically representative and multi-racial/ethnic samples is warranted to ensure its applicability across diverse populations. Second, we defined central obesity using international WC 90th percentile values rather than individual population-specific 90th WC references (in consideration of the variation of WC across countries/races). We did this just for the direct comparison between countries using a unified international WC reference. However, the validation should be conducted based on population-specific WC references in future when individual population-specific WC references are available. Third, we just assessed the application of pediatric MetS in adolescents aged 12–17 years, as the IDF or NCEP definition was only recommended to be applied in adolescents aged ≥ 10 years or ≥ 12 years, and there is no “gold standard” for children aged < 10 years which we can use for the validation. Future research also should assess the performance of simplified definition in children aged 6–11 years, and subclinical vascular damage may be the optimal outcome to assess the impact of MetS in younger children. Fourth, our study was cross-sectionally designed and causality inference should not be made. Further rigorous epidemiological studies including prospective follow-up study and even clinical implementation are warranted to assess the utility and long-term prognostic value of MetS risk estimated by the proposed simplified definition in predicting future cardiovascular outcomes later in life. Fifth, the practicality of assessing multiple components in routine pediatric care settings is still insufficient, as the current definition of pediatric MetS requests at least three of five components according to the IDF or NCEP definition. Future studies should consider this challenge.

## Conclusions

The proposed simplified definition with two risk assessment levels may be useful for pediatricians to quickly identify severity of MetS risk and CMRFs clustering in clinical practice. A simple and consistent definition that involves static cut-offs for assessing MetS risk in pediatric population may allow robust comparison of MetS prevalence and consistent risk monitoring across different pediatric populations globally. Additionally, our study also highlight that future research should address methodological limitations, explore long-term prognostic value of MetS risk estimated using the proposed definition, and assess whether this proposed definition can optimize clinical implementation strategies.

### Supplementary Information

**Additional file 1: Table S1.** Prevalence of the MetS and its components based on the simplified definition in 10 pediatric populations. **Table S2.** Comparison of MetS prevalence between the simplified definition and the IDF or NCEP definition in 10 pediatric populations. **Table S3.** Comparison of MetS prevalence between the simplified definition with or without central obesity as an essential component in 10 pediatric populations.

## Data Availability

The data that support the findings of this study are available from the corresponding author upon reasonable request.

## Abbreviations

AUC: Area under the curve
BMI: Body mass index
CI: Confidence interval
CMRFs: Cardiometabolic risk factors
DBP: Diastolic blood pressure
FBG: Fasting blood glucose
HDL-C: High-density lipoprotein cholesterol
IDF: International Diabetes Federation
MetS: Metabolic syndrome
NCEP: National Cholesterol Education Program
NHANES: National Health and Nutrition Examination Survey
NPV: Negative predictive value
OR: Odds ratio
PPV: Positive predictive value
ROC: Receiver operator characteristic
SBP: Systolic blood pressure
TG: Triglycerides
USA: United States of America
WC: Waist circumference
WHtR: Waist-to-height ratio
