# Supplementary material for: A proposed simplified definition of metabolic syndrome in children and adolescents: a global perspective
Source: BMC Med. 2024 May 7;22:190. doi: 10.1186/s12916-024-03406-y (PMC11077757; doi:10.1186/s12916-024-03406-y)
Supplement: Supplementary file 1 — Additional file 1: Table S1. Prevalence of the MetS and its components based on the simplified definition in 10 pediatric populations. Table S2. Comparison of MetS prevalence between the simplified definition and the IDF or NCEP definition in 10 pediatric populations. Table S3. Comparison of MetS prevalence between the simplified definition with or without central obesity as an essential component in 10 pediatric populations. [file 12916_2024_3406_MOESM1_ESM.doc]

**Table S1 Prevalence of the MetS and its components based on the simplified definition in 10 pediatric populations**

| Country | Survey year | Age  (years) | Sample size | Boys, n (%) | Overweight &obesity,  n (%) | Central obesity, n (%) | High BP,  n (%) | High TG,  n (%) | Low HDL-C,  n (%) | High FBG,  n (%) | MetS, n (%) |
| --- | --- | --- | --- | --- | --- | --- | --- | --- | --- | --- | --- |
| Brazil_a | 2012-2013 | 15-17 | 441 | 141 (32.0) | 72 (16.3) | 101 (22.90) | 32 (7.26) | 42 (9.52) | 181 (41.04) | 0 | 17 (3.85) |
| Brazil_b | 2014-2016 | 12-17 | 392 | 213 (54.3) | 90 (23.0) | 75 (19.13) | 14 (3.57) | 13 (3.32) | 84 (21.43) | 22 (5.61) | 6 (1.53) |
| China | 2009 | 12-17 | 386 | 204 (52.8) | 39 (10.1) | 79 (20.47) | 53 (13.73) | 73 (18.91) | 46 (11.92) | 29 (7.51) | 22 (5.70) |
| Germany | 2000-2007 | 12-17 | 2780 | 1505 (54.1) | 421 (15.1) | 317 (11.40) | 475 (17.09) | 122 (4.39) | 236 (8.49) | 818 (29.42) | 84 (3.02) |
| Greece | 2008-2010 | 12-17 | 379 | 176 (46.4) | 151 (39.8) | 189 (49.87) | 100 (26.39) | 36 (9.50) | 38 (10.03) | 68 (17.94) | 43 (11.35) |
| Iran | 2011-2012 | 12-17 | 5014 | 2510 (50.1) | 769 (15.3) | 1379 (27.50) | 966 (19.27) | 816 (16.27) | 1838 (36.66) | 424 (8.46) | 427 (8.52) |
| Italy | 2007-2008 | 12-13 | 374 | 193 (51.6) | 148 (39.6) | 178 (47.59) | 36 (9.63) | 24 (6.42) | 59 (15.78) | 6 (1.60) | 16 (4.28) |
| Korea | 2001-2013 | 12-17 | 5240 | 2776 (53.0) | 1134 (21.6) | 1268 (24.20) | 461 (8.80) | 728 (13.89) | 1004 (19.16) | 477 (9.10) | 330 (6.30) |
| South Africa | 2007-2008 | 12-16 | 989 | 396 (40.0) | 229 (23.2) | 274 (27.70) | 116 (11.73) | 61 (6.17) | 528 (53.39) | 39 (3.94) | 56 (5.66) |
| USA | 2001-2018 | 12-17 | 3431 | 1747 (50.9) | 1320 (38.5) | 1204 (35.09) | 176 (5.13) | 364 (10.61) | 405 (11.80) | 503 (14.66) | 205 (5.97) |
| Total | 2000-2018 | 12-17 | 19426 | 9861 (50.8) | 4373 (22.5) | 5064 (26.07) | 2429 (12.50) | 2279 (11.73) | 4419 (22.75) | 2386 (12.28) | 1206 (6.21) |

*Abbreviations BP* blood pressure, *FBG* fasting blood glucose, *HDL-C* high-density lipoprotein cholesterol, *MetS* metabolic syndrome, *TG* triglycerides, *USA* United States of America.

Overweight and obesity were defined according to BMI categories using Cole’s cut-off points [25].

**Table S2 Comparison of MetS prevalence between the simplified definition and the IDF or NCEP definition in 10 pediatric populations**

| Country | Sample size | IDF definition, n(%) | NCEP definition, n(%) | Simplified definition, n(%) | Simplified definition *versus* IDF definition | |  | Simplified definition *versus* NCEP definition | |
| --- | --- | --- | --- | --- | --- | --- | --- | --- | --- |
|  |  |  |  |  | Chi-square | *P*-value |  | Chi-square | *P*-value |
| Brazil_a | 441 | 17 (3.85) | 30 (6.80) | 17 (3.85) | 0 | 1.000 |  | 13.000 | 0.0003 |
| Brazil_b | 392 | 5 (1.28) | 8 (2.04) | 6 (1.53) | 1.000 | 0.3173 |  | 0.667 | 0.4142 |
| China | 386 | 12 (3.11) | 16 (4.15) | 22 (5.70) | 10.000 | 0.0016 |  | 3.000 | 0.0833 |
| Germany | 2780 | 85 (3.06) | 147 (5.29) | 84 (3.02) | 0.014 | 0.9055 |  | 31.752 | <0.0001 |
| Greece | 379 | 34 (8.97) | 56 (14.78) | 43 (11.35) | 4.263 | 0.0389 |  | 4.122 | 0.0423 |
| Iran | 5014 | 197 (3.93) | 442 (8.82) | 427 (8.52) | 188.929 | <0.0001 |  | 0.818 | 0.3657 |
| Italy | 374 | 11 (2.94) | 31 (8.29) | 16 (4.28) | 2.778 | 0.0956 |  | 13.235 | 0.0003 |
| Korea | 5240 | 221 (4.22) | 411 (7.84) | 330 (6.30) | 80.823 | <0.0001 |  | 32.320 | <0.0001 |
| South Africa | 989 | 35 (3.54) | 77 (7.79) | 56 (5.66) | 14.226 | 0.0002 |  | 7.737 | 0.0054 |
| USA | 3431 | 190 (5.54) | 284 (8.28) | 205 (5.97) | 2.647 | 0.1037 |  | 39.752 | <0.0001 |
| Total | 19426 | 807 (4.15) | 1502 (7.73) | 1206 (6.21) | 240.849 | <0.0001 |  | 96.706 | <0.0001 |

*Abbreviations IDF* International Diabetes Federation, *MetS* metabolic syndrome, *NCEP* National Cholesterol Education Program, *USA* United States of America.

**Table S3 Comparison of MetS prevalence between the simplified definition with or without central obesity as an essential component in 10 pediatric populations**

| Country | Sample size | MetS prevalence based on simplified definition, n (%) | | Chi-square | *P*-value |
| --- | --- | --- | --- | --- | --- |
|  |  | Central obesity is not an essential component | Central obesity is an essential component |  |  |
| Brazil_a | 441 | 17 (3.85) | 17 (3.85) | — | — |
| Brazil_b | 392 | 6 (1.53) | 6 (1.53) | — | — |
| China | 386 | 22 (5.70) | 20 (5.18) | 2.000 | 0.1573 |
| Germany | 2780 | 84 (3.02) | 61 (2.19) | 23.000 | <0.0001 |
| Greece | 379 | 43 (11.35) | 41 (10.82) | 2.000 | 0.1573 |
| Iran | 5014 | 427 (8.52) | 374 (7.46) | 53.000 | <0.0001 |
| Italy | 374 | 16 (4.28) | 16 (4.28) | — | — |
| Korea | 5240 | 330 (6.30) | 305 (5.82) | 25.000 | <0.0001 |
| South Africa | 989 | 56 (5.66) | 53 (5.36) | 3.000 | 0.0833 |
| USA | 3431 | 205 (5.97) | 194 (5.65) | 11.000 | 0.0009 |
| Total | 19426 | 1206 (6.21) | 1087 (5.60) | 119.000 | <0.0001 |

*Abbreviations MetS* metabolic syndrome, *USA* United States of America.
